# The Role of Gut Microbiota in Different Types of Physical Activity and Their Intensity: Systematic Review and Meta-Analysis

**DOI:** 10.3390/sports12080221

**Published:** 2024-08-14

**Authors:** Tehreema Ghaffar, Francesca Ubaldi, Veronica Volpini, Federica Valeriani, Vincenzo Romano Spica

**Affiliations:** Department of Movement, Health and Human Sciences, University of Rome “Foro Italico”, 00135 Rome, Italy; t.ghaffar@studenti.uniroma4.it (T.G.); f.ubaldi@studenti.uniroma4.it (F.U.); v.volpini@studenti.uniroma4.it (V.V.); vincenzo.romanospica@uniroma4.it (V.R.S.)

**Keywords:** gut microbiota, physical activity, physiological factors, diet, athletes

## Abstract

Background. Intense exercise during training requires dietary modulation to support health and performance and differs in different types of activities. Diet, supplementation with prebiotics and probiotics, and, more recently, even physical activity can potentially improve health outcomes by modifying and protecting the gut microbiota. A systematic review and meta-analysis were conducted to investigate the modulation of gut microbiota in different types and intensities of physical activity and different lifestyles of athletes. Methods. The systematic review and meta-analysis were conducted according to the PRISMA guidelines, and the protocol was registered in PROSPERO (CRD42024500826). Results. Out of 1318 studies, only 10 met the criteria for inclusion in the meta-analysis. The pilot study’s meta-regression analysis highlights the role of type and intensity of exercise in changing the B/B (Bacillota/Bacteroidota) ratio (*p* = 0.001). Conclusions. As gut training becomes more popular among athletes, it is necessary to map interactions between microbiota and different types of physical activity, personalized diets, physical activities, and ergogenic supplements to enhance performance and athletic wellness.

## 1. Introduction

The human gut microbiota is the population of microbes, including bacteria, archaea, eukaryotes, and viruses that inhabit our gastrointestinal tract [1]. It has coevolved with humankind and is thought to number approximately 100 trillion [1,2]. The main bacteria taxa are in different relative amounts, such as Bacillota, Bacteroidota, Cyanobacteria, Pseudomonadota, Fusobacteria, Actinomycetota, and Verrucomicrobiota. The first two phyla represent about 90% of the bacterial flora population in the gut, especially the colon [3,4]. Gut microbiotas possess metabolic, protective, and structural functions in the gut mucosa [5]. Over the years of research and development of sequencing technologies and projects such as the Human Microbiome Project (HMP), the knowledge of the human microbiome has increased as well as the awareness about the host–microbial flora interaction influencing the health status of humans [6]. The gut microbiota matures during the first years of life, but its development depends upon several factors like the mode of birth, dietary interventions, intake of antibiotics, and the interference of some environmental factors including exercise, diet, and stress that can lead to a state of eubiosis or dysbiosis [7].

Scientific evidence shows that these groups of organisms are not only involved in local processes such as maintaining the balance of the mucous membranes and preserving the integrity of the epithelial cells, as well as in the production and absorption of nutrients, but they also play a role in the immune system and the nervous system, forming part of the brain–gut axis [4,8]. In particular, gut microbes can change the homeostasis of the host by producing vitamins, short-chain fatty acids, and amino acids starting from food components [9,10]. Sport and physical activity, including leisure activities, planned exercise, and various sports disciplines, are essential for maintaining a healthy lifestyle and are fundamental tools in preventing and treating many chronic diseases by influencing the intestinal microbiota [11]. 

Research shows that certain groups of microorganisms, such as SCFA producers, are associated with exercise. These microorganisms may play a role in maintaining the balance of the intestinal lining, increasing mucus thickness, improving the body’s metabolic and immune functions, and influencing the connection between the gut and the brain. As a result, they may help reduce neuroinflammation and mental fatigue [12,13,14]. Exercise intensity determines the effect of gut microbial diversity as low-intensity exercise reduces the transit time of stool and ultimately reduces the connection time between the microbiota and mucus layer. Endurance exercises lead to changes in the gastrointestinal tract, leading to reduced splanchnic blood flow to almost 80% of the basal level [15]. Prolonged exercises possess beneficial effects like bacterial translocation from the colon, increased intestinal permeability, and a compromised gut barrier [5]. 

The intensity of the physical activity modulates the gut microbiota and a number of short-chain fatty acids. Aerobic exercises performed for at least 60 min and 60% HRmax per day tremendously increased the beta diversity indexes of gut microbiota and SCFAs [16]. The gut microbiota is influenced by the physical activity and the dietary intake of athletes, collectively influencing physical performance, so boosting the athletes’ capabilities is essential to fuel the gut bacteria through diet and exercise. Especially high fiber diets, protein-rich foods, unsaturated fats, and supplements have a positive impact [17]. The endurance exercise impact gut bacteria depending on the subject and its intensity. It was found that in athletes, endurance exercises depict a negative effect on the intensity of gut bacteria, while in non-athletes, this effect was less as compared to athletes [18].

Exercise-related stress might be associated with changes in the structure of the intestinal microbiota. The evaluation of associations among gut microbiota and diet and exercise in different athletes such as bodybuilders and elite runners was considered. The results indicate differences in gut microbiota diversity according to athlete, sport type, and diet [19]. Also, experimental studies on animal models have accessed the interaction between gut microbiota and exercise intensity. The stool samples of animal models collected at different intervals were analyzed using 16S amplicon sequencing, and moderate-intensity exercise seems to affect both the gut microbiota and metabolism [20]. An observational trial recruiting elite athletes performing endurance training and consuming high protein and high carbohydrate diets showed how the dietary intake may impact gut flora with implications on the performance [21]. 

The athlete’s gut microbiota differs significantly from non-athletes in terms of both composition and metabolic functioning [22]. Physical activity is linked with a tremendous increase in gut microbial diversity and an increment in the yield of short-chain fatty acids by bacteria which leads to an increase in butyrate production. The SCFAs have a role in maintaining homeostasis, epithelial integrity, mental fatigue, and neuroinflammation. 

Scientific evidence differs in several issues, such as the kind of biodiversity change and the role of dietary behavior vs. physical activity or other variables [13,23,24]. A systematic review conducted in 2021 shows that most of the included papers registered greater variability and frequency of the phylum Bacillota in active versus sedentary subjects. Furthermore, studies that have performed dietary control have reported associations between physical activity and some bacterial families, including Lachnospiraceae, Clostridiaceae, Paraprevotellaceae, Ruminococcaceae, and Veillonellaceae [23]. An experimental study conducted on young Caucasian adults also showed a positive correlation of the phylum Bacillota with BMI [24]. However, it is necessary to further investigate how specific foods or supplements can modulate the possible influence of exercise on microbial diversity in the gut [13].

Furthermore, human studies, at least to date, have a heterogeneous model design, approaching different kinds, doses, and durations of exercise [9]. Experimental studies revealed a strong relationship between microbiota, diet, sport, health, and athletic performance [25]. Given the vast diversity and number of gut microbiota and their interaction with exercise, the data may not always be current or authentic, making the literature on the role of microbiota seem scarce [25,26,27,28]. Nonetheless, existing data predicts that the microbiome can be influenced positively or negatively, depending on specific dietary intake, type, intensity, and duration of exercise. This implies that a deeper investigation into the role of individual species could lead to strategies designed to promote beneficial gut microbiota and suppress harmful ones. Therefore, it is crucial to expand our understanding of how gut microbiota interact with various types of exercise [28]. This review seeks to examine how different types and intensities of physical activity affect microbiota diversity.

## 2. Materials and Methods

### 2.1. Selection Protocol and Search Strategy

The present review was conducted according to the PRISMA guidelines [29]. The protocol was registered in PROSPERO (Reference Number CRD42024500826). PICOS frame and the specific eligibility criteria we used to perform this review. Especially, the criteria are (a) population health subjects; (b) intervention of any type of physical activity; (c) comparison not physical conditioning or other types of physical training; (d) outgrowth assessment of differences for α-diversity and β-diversity, relative diversity of specific bacteria, and/or metagenomic data anatomized with 16S amplicon sequencing; (e) papers written in English; (f) observational studies (cross-sectional, retrospective, and prospective studies) and experimental study design. Clinical trials, reviews, meta-analyses, case studies, case reports, proceedings, qualitative studies, commentary studies, and studies without a control group, as well as studies with unhealthy subjects and incomplete designs, were excluded. PubMed, ScienceDirect, and Scopus databases were accessed using the following terms (“gut microbiota, physical activity” [MeSH Terms] OR (“microbiota” [All Fields] AND “physical activity” [All Fields] AND “diet” [All Fields]) OR “human gut microbiota” [All Fields] OR (“microbiota” [All Fields] AND “Gut” [All Fields] AND “physical activity” [All Fields])) AND (“microbiota” [MeSH Terms] OR “microbiota” [All Fields] OR “microbiotas” [All Fields] OR “microbiotas” [ All Fields] OR “microbiota” [All Fields] OR “microbiome”). The search string used on PubMed was carried out by title, abstract, and MeSH terms; the hunt on Scopus and Web of Science involved content by the title, abstract, and keywords. The search was conducted until April 2023. The screening of studies consisted of a multi-step rejection process, with four experts personally reviewing the titles and abstracts of the studies [29]. The titles and abstracts gathered from these three databases were then imported into covidence—a tool for better systematic review management—to facilitate the assessment of their relevance [30]. The coming step was screening by title and abstract the possibly eligible studies, following the additional criteria mentioned ahead; this step was performed by 4 authors (T.G., F.V., F.U., V.V.) singly. Also, full textbooks were read singly by the 4 authors (T.G., F.V., F.U., V.V.) with a discussion about their addition in the review. Dissensions were achieved by agreement among the authors. 

### 2.2. Data Extraction Process and Quality Assessment 

The data extracted from each eligible record included bibliographical information, design of the study, population characteristics, type of exercise, duration, frequency, training volume, and microbial profile analysis. 

Adequate quality assessment tools for every type of study comprised in the review were used to assess the quality of eligible articles. Cross-sectional studies were calculated by the New Castle–Ottawa Quality adapted for cross-sectional studies. Cohort studies were assessed by the Newcastle–Ottawa Scale for Cohort Studies (NOS) [31,32,33]. A comprehensive quality rating was assigned to every eligible article depending on the number of values met as follows: good quality (all values met, low risk of bias); fair quality (1 value not met or 2 values unclear, moderate risk of bias); poor quality (2 or more values not met, high risk of bias). Randomized clinical trials were evaluated by the Cochrane collaboration risk-of-prejudice tool for randomized trials (RoB2) [34]. Each RCT was graded according to five domains: randomization, deviation, missing data, outcome measurement, and selective reporting, and an overall score for risk of bias was assigned. Four reviewers (T.G., F.U., V.V., F.V.) independently assessed the risk of prejudice for the articles comprised, according to the four authors singly assigned a score to each study, and conflicts were settled by agreement among all the authors. 

### 2.3. Data Synthesis 

For differences in the overall content of the gut microbiota, we only performed a descriptive literature fusion because of the different situations of determination for the proportion of microbiota, the small sample sizes, and the insufficient data and quality of the involved studies. We also performed meta-analyses by using comprehensive meta-analysis [35]. The Bacillota/Bacteroidota (B/B) ratio, which is commonly used to assess gut health, was one of the indices considered in this study. In cases where there were issues with the unit of analysis, only the total number of participants in the control group were divided, and the means and standard deviations remained unchanged. The 95% confidence interval (95% CI) was also calculated. Effect size was distributed as small (SMD = 0.2), moderate (SMD = 0.5), and large (SMD = 0.8) [35]. The statistic test was used to assess the diversity of named studies and testing the classical measure of diversity is Cochran’s Q (Hedges Q statistic). The thresholds test for the interpretation of I_2_ were as follows: <25%, low diversity; <50%, moderate heterogeneity; and >75%, high diversity. To estimate the publication bias, due to the high volume of samples included, the Egger’s test and channel plot were performed. Subgroup analyses were carried out for outcomes reported in studies with two or more groups within each subgroup. Meta-regression and subgroup analysis were used to find the expected sources of heterogeneity [36]. Predefined subgroup analyses were performed, including chronic or acute intervention, type of aerobic or anaerobic, intensity of physical activities, sample size, ages, publication year, and methodological quality of the study. For physical activity intensity, we used different classifications, based on a percentage of maximal aerobic power (VO_2_ max and VO_2_R) and metabolic equivalents (METs) values [37,38,39]. To perform a sensitivity analysis, we either excluded individual studies or modified the effects model [35,36,40].

## 3. Results

The identification of the specific physical activity is required to identify the strains of gut microbiota in the specimens. The purpose of determining the strains of bacterial species is to find the alterations in the gut biodiversity depending on the type and intensity of physical exercise [41,42]. In this context, the knowledge of microbiota structure and its modification is fundamental to drawing these interventions. Here, we aim to summarize the data on the microbiota diversity in athletes depending on their activity and how the different types of exercises can modify their composition.

**Figure 1:**
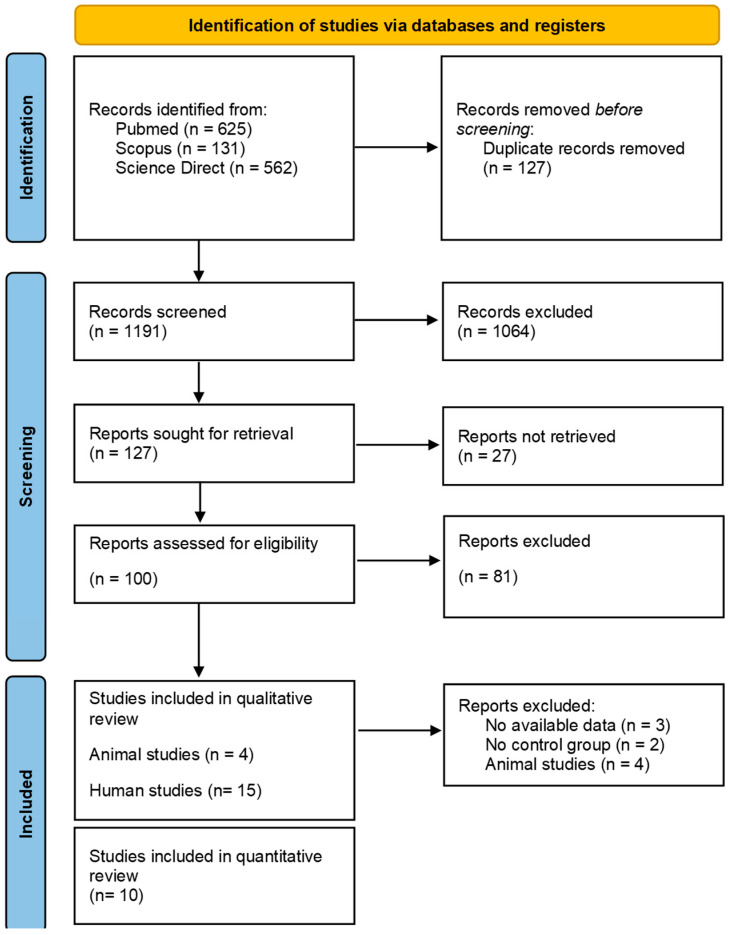
PRISMA flow diagram following the PRISMA guidelines [29,40].

Figure 1 shows the steps of the selection procedure for the systematic review [29,40]. In Table S1, the PRISMA checklist shows the location where the item is reported in the paper. In total, 1318 studies were retrieved from all searched databases and, after expelling duplicates, 127 articles remain for the following steps. Of the remaining studies, 100 were selected for full-text analysis. Then, the full texts of selected articles were accessed and evaluated, considering the inclusion and exclusion criteria. After the assessment, 81 articles were excluded based on the exclusion criteria. Finally, 19 articles fulfilled the inclusion criteria and were placed in the systematic review [42,43,44,45,46,47,48,49,50,51,52,53,54,55,56,57,58,59,60], and 10 articles were used for meta-analysis [43,46,50,51,53,54,55,56,58,59].

Based on the results collected from the previously available narrative reviews, a meta-analysis was conducted, focusing on a few studies that could be covered in the systematic review. The studies enclosed in the systematic review are divided into two groups, one group contains all the animal studies (Table 1) [42,44,45,46], and the other group includes all the human studies (Table 2) [43,47,48,49,50,51,52,53,54,55,56,57,58,59,60].

The articles involved in the systematic review focusing on animal studies were published between 2017 [42] and 2021 [44,45] and show a very stable trend regarding the research in this field. One article was published in 2017 [42], one in 2019 [46], and two studies were published in 2021 [44,45]. The research in the animal field depicts a global dimension as the studies were conducted in various countries including one study in Finland [42], one study in the USA [43], one study in Taiwan [46], one study in China [44], and one study conducted in France [45]. In the systematic review papers, the specific study design was used to evaluate the results of all four studies conducted. On animals, the cohort study design was used to access the results [42,44,45,46]. The sample size used in the articles ranges from 20 subjects [42,45], to 30 subjects [44], to 100 subjects [46], and for animal studies, a wide range is required to access good results. The age number of the subjects ranges from 5 weeks [44] to 12 years [45], so it is a good factor to evaluate results in the small age group vs. mature groups. The subjects are 80% male and 20% female. Different physical exercises studied in the systematic review papers considering animals include exercises like exhaustive swimming in which weight is tied to the tail of mice to produce exhaustion during swimming [46]. In another study endurance and treadmill exercises were performed collectively to access the combined effect [42]. One study evaluated the results of endurance exercise on the gut microbiota [45] and the last study assessed treadmill exercises [44]. Thus, all these studies evaluated the results of these exercises in gut microbiota changes. 

For the human studies, all the included articles were published between 2017 [43,47,48] and 2021 [49,50,51,52] and these studies were performed in several countries and show a very stable increasing trend. Three studies were published in China [48,53,54], two in the USA [43,48], one in Ireland [55], one in Poland [56], one in France [49], one in Portugal [50], one in Australia [51], one in New Zealand [57], one study in Canada [58], one in German [52], one in Korea [59], and one in Ireland [60]. The research related to the gut microbiota and its interaction with different types of physical activities has increased tremendously in recent years, showing a positive trend and interest in these terms depending on the health sector. 

Different types of study designs used in the papers were included in the systematic review. The study design includes three randomized controlled designs [47,52,55], seven cross-sectional studies [43,48,54,56,57,58,59], four cohort studies [50,51,53,60], and one clinical trial [43]. In all the papers, the sample size ranges from 2 [60] to 90 [55], which is a very good ratio as the lowest and highest ranges are included in it. For the systematic review papers, the subjects included in these studies range from 12 years [53] to 72 years [56]. This impact of physical activity on gut microbiota covers a maximum age gap to deliver all the possible effects depending on the age groups. In total, 60% of the subjects in these articles are male, and 40% of the subjects are female. 

For quality assessment, cross-sectional studies [43,48,54,56,57,58,59] were calculated by the New Castle–Ottawa Quality adapted for cross-sectional studies and the score was 83% fair with moderate risk of bias [48,54,57,58,59]. Cohort studies [42,44,45,46,49,50,51,53,60] were assessed by the Newcastle–Ottawa Scale for Cohort Studies (NOS) and the results showed low-risk bias in 45% of articles [42,49,51,53] and moderate risk in 55% [44,45,46,50,53,60]. Randomized clinical trials [47,52,55] were evaluated by the Cochrane collaboration risk-of-prejudice tool for randomized trials (RoB2) and the results showed a low-risk bias in two papers [52,55].

There are several types of physical activities and each type then alters the gut microbiota in its own way, so the articles included in the systematic review cover several types of athletes and sports including running [49,51,56,59], cycling [43] strength and endurance exercises [52], bodybuilding [59], rowing [53], skiing [56], football [50], aerobic and resistance training [55], martial arts [54], 51 km cross-country ski-march [47], and HIIT (high-intensity interval training) [57]. So, these studies presented a broad spectrum to evaluate diversity including all the possible physical activities. 

Table 2 presents the papers encompassed in the meta-analysis, outlining the microbiota profiles and alterations resulting from physical training. It also classifies the levels of physical activity intensity for each group, control and experimental, across all studies. 

**Figure 2:**
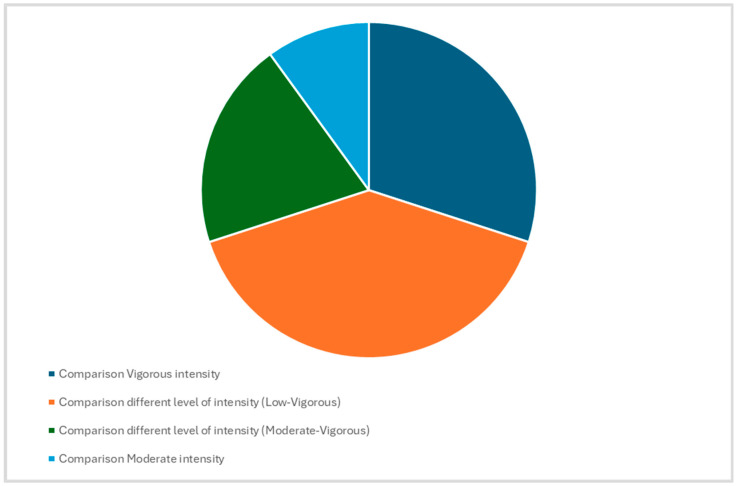
The pie chart illustrates the design of studies and the different levels of intensity investigated categorized by exercise intensity levels (low, moderate, vigorous). Specifically, for categorization, the following intensity levels were considered: low intensity (less than 3 METs or less than 30% of VO2 max); moderate intensity (between 3 and 6 METs or between 30% and 60% of VO2 max); vigorous intensity (more than 6 METs or more than 60% of VO2 max) [37,38,39].

In the pie chart, the designs of studies and the different levels of intensity investigated categorized by exercise intensity levels (low, moderate, vigorous) were summarized (Figure 2). These findings show the current research trends. In 30% of the studies included in the meta-analysis, authors performed the comparison between the same intensities [43,47,51,54], and in the 60%, the comparison was carried out between different levels of intensity [50,53,55,56,58,59], specifically in 40% low (less than 30% of VO_2_ max) versus vigorous (more than 60% of VO_2_ max), and 20% moderate (between 30% and 60% of VO_2_ max) versus vigorous (more than 60% of VO_2_ max).

The 10 studies selected for meta-analysis were performed on humans [43,47,50,51,53,54,55,56,58,59]. In 80% of the studies, fecal analysis is conducted after chronic adaptations from specific regular training over time [43,50,51,53,54,55,56,59], while the remaining 20% of analyses are performed post-training (immediately or until 1 week) [47,58]. All the studies were conducted on fecal samples using the Miseq platform Illumina technique, except one study [56] in which an ion torrent PGM sequencer was used. The V3–V4 regions of 16S amplicon sequencing are used in all the works, but different primers including the sequencing and universal primers are used. In all of the ten papers admitted in the meta-analysis, alpha and beta diversity was used to determine biodiversity, and in the eighth [43,47,50,51,53,54,56,58] of the ten papers, the Shannon index increased in the population performing high-intensity physical activities. They seem not modified in the presence of different aerobic physical activities or intensity. 

The ratio, the B/B ratio index, was enhanced in subjects with physical activity intensities [53,56,59]. Specifically, in 50% of the studies (5 out of 10), there was an increase in the B/B ratio, and in four of these cases, it was associated with an increase in the intensity of training, ranging from low or moderate to vigorous, or with an increase in the volume of exercise load (hours per week) [43,47,53,56,59].

**Figure 3:**
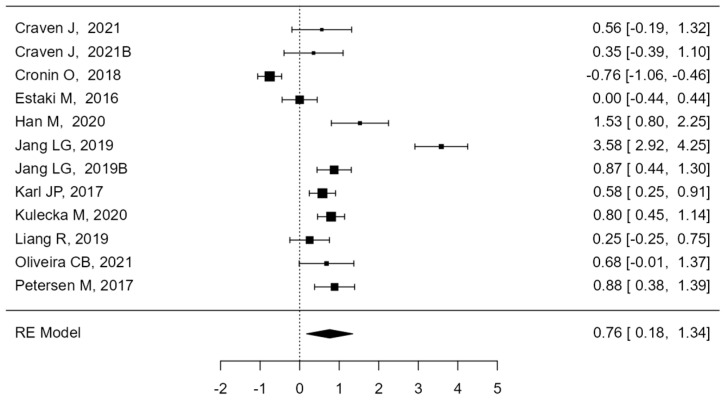
The results of the meta-analysis are shown in forest plot: the differences in the means of the Bacillota/Bacteroidota ratio for each study are analyzed [43,47,50,51,53,54,55,56,58,59].

Further analysis using the stratification way of intensity of physical activity data (Figure 3) verified this trend. The meta-analysis was performed using the standardized mean difference as the results measure. A total of 10 studies were comprehended in the analysis, but two study are considered two different populations. The observed standardized mean differences ranged from −0.7583 to 3.5830, with most estimates being positive (83%). The estimated average standardized mean difference entrenched on the random-effects model was $\hat{\mu }$ = 0.7597 (95% CI: 0.1764 to 1.3429). Therefore, the standard outcome varied significantly from zero (z = 2.5527, *p* = 0.0107). According to the Q-test, the true results appear to be heterogeneous (Q(11) = 170.5522, *p* < 0.0001, tau^2^ = 0.9827, I^2^ = 94.4979%). A 95% prediction interval for the actual results is given by −1.2689 to 2.7882. Hence, although the average result is approximated to be positive, in some studies, the true results may in fact be negative.

The Bacillota/Bacteroidota ratio (formerly Firmicutes/Bacteroidetes), which describes gut health, was basically analyzed. In fact, it has been recommended as an achievable biomarker of dysbiosis since it has been described by several studies that there is a difference in this index among normal weight and obese individuals. Consequently, the B/B ratio is often cited in the scientific literature as an indication of obesity or less-than-good health states, although the scientific community is not unaware of this definition. In the present study, as shown in Table 3 and Figure 3, the B/B ratio changed based on how various levels of intensity of exercise impacted the gut microbiota ratios.

An examination of the residuals revealed that one study [59] had a value larger than ±2.8653 and maybe a potential outlier in the context of this model, which could be overly influential [59]. There was no evidence of funnel plot asymmetry in either the regression test or the rank correlation (*p* = 0.1256 and 0.6384, respectively; Figure S1). Finally, to find the effect of moderator variables on the B/B ratio, subgroup analyses and me-ta-regressions were performed. We performed a meta-regression of the incorporated intervention types (type—aerobic or anaerobic, intensity or duration), sample size, the publication year, the methodological quality of the study, and the WHO region origin. The meta-regression only revealed that the type of treatment had an impact on the Bacillota/Bacteroidota ratio in adults (*p* = 0.004) and the intensity of exercise determines these ratios. The meta-regression revealed that the type of treatment had an impact on the B/B ratio in adults (*p* = 0.004) and the intensity of exercise determines these ratios. This was also seen, but only if associated with vigorous intensity, for the duration (*p* = 0.410).

## 4. Discussion

The importance of the gut microbiota on human health and sports performance has come to light in recent decades. Different physical activity patterns can have different effects on the gut microbiota and, in turn, on athletic performance. Here, in this systematic review and meta-analysis we examine how various forms and intensity levels of physical activity and their impact on microbial diversity. 

Specifically, in the qualitative systematic review emerge different important aspects from the included studies. In their 2021 study, Moitinho–Silva et al. [52] explored the interaction of different physical activities like strength exercises and endurance exercises and found that typologies of exercise have different but moderate impacts on the overall human physiology and very distinct microbial modifications in the gut. They involve subjects practicing endurance and strength training compared to controls who did not exercise regularly. As a result, the physically active group shows significantly more of the genera *Coprococcus*, *Parasutterella,* and the family *Ruminococcaecae* in comparison to the control group. In 2019, Jang et al. [59] performed a cross-sectional study using bodybuilders and distance runners, providing a high-protein, high-fat, low-carbohydrate, and low-fiber diet to bodybuilders and a low-carbohydrate and low-fiber diet to distance runners. The athletes consuming a high-protein and low-carbohydrate diet had decreased diversity of gut microbiota and decreased abundance of short-chain fatty acid-producing bacteria, which depicts the role of diet alongside physical activity to affect gut microbial diversity. In 2017, Karl et al. investigated military training effects using a randomized controlled trial in three different groups, provided with protein-based and carbohydrate-based diets and 4 days arctic military training [47]. They observed that physiological stress influences intestinal permeability and modifying diet changes gut microbiota composition. Moreover, the Shannon and chao1 indices showed an increasing trend [47].

In 2020 cohort research conducted by Han et al. [53], the gut microbial communities of elite and non-elite athletes at the juvenile and adult levels were observed to see how they vary on the type of exercise in relation to nutritional variables and physical attributes. Kulecka et al. [56] conducted a cross-sectional study on cross-country skiers and marathon runners who had undergone the most intense endurance training. They found that intensive training is linked to both compositional changes and the growth of greater bacterial diversity. Additionally, athletes’ enhanced taxa are known to be involved in the fermentation of fiber. In 2017, Yang et al. conducted a cross-sectional study [48] wherein premenopausal women were assessed for VO_2_-max. The findings indicated that gut microbiota composition and cardiorespiratory fitness are related, independent of age and carbohydrate or fat intake. The relationship between VO_2_-max and gut microbiota, on the other hand, seems to be mediated by body fatness and physical activity.

In 2021, Tabone et al. [49] performed a clinical trial on cross-country runners and involved them in performing different treadmill exercises at different intensities, the results depict that the performance of an individual exercise bout, in cross-country non-professional athletes, shows significant changes in the microbiota, in serum and fecal metabolome, which may have the health implications. In 2021 Oliveira et al. [60] conducted a cohort study in which the perceived exertion method was applied on female football players to observe the changes in gut microbial diversity before and after the football tournament. The gut microbiota composition of elite female footballers was not altered by the physical and physiological demands of training and matches in an official international tournament. Although the Shannon, chao1, and Simpson indices increased and the Bacillota and Bacteroidota did not change much before and after the tournament. In 2018, Cronin et al. [55] observed in athletes performing aerobic and resistance exercise training for 8 weeks that the enhanced body composition with exercise does not rely on major changes in the composition of microbial populations in the gut. 

In 2021, Barton et al. [60] performed a 6-month cohort study on male marathon and triathlon runners, involving them in regular aerobic exercise complimented with twice-weekly resistance training, and observed alterations in gut microbiota and physiologically relevant metabolites due to sustained fitness exercises. In 2021, Craven et al. [61] conducted a 7-week cohort study on middle-distance runners and different training volumes to observe the association with gut microbiota. The results revealed that the alpha-diversity and global composition of the gut microbiome were unchanged by modifications in training volume. 

In 2021 [57], Rettedel et al. performed a cross-sectional study in which 3 weeks of HIIT training were used to observe the influences on intestinal microbiota. Short-term HIIT does not impact the gut microbiome, but some genera are linked with some metabolic process in participants. 

In 2016, Estaki et al. conducted a cross-sectional study and observed a correlation between improved cardiovascular fitness and greater microbial diversity in healthy humans, with the associated alterations anchoring around a set of functional nuclei rather than a particular taxonomic group [58]. In 2019, Liang et al. [54] performed a cross-sectional study on two groups of martial art athletes, group 1 performing high-level exercise and group 2 with low-level exercise. They were observed for any changes in gut microbiota depending upon the intensity of physical activity. The results indicate the increased diversity and higher metabolic capacity of the gut microbiome in group 1 as compared to group 2. According to Karl et al. [47], a good increasing trend on the ratio of Bacillota to Bacteroidota and increasing trend in proteobacteria and actinobacteria were observed. Also, stress was associated with intestinal permeability and changes in gut microbiota by modifying diet. In a study conducted by Han in 2020, the gut microbial communities depend upon the type of exercise associated with dietary factors and physical characteristics [53]. In 2021, Mach et al. [45] performed a study on horses, in which he modulated the level of physical activities and targeted the gut-mitochondria axis, and it appears to be a potential strategy to enhance horses’ physical performance. 

**Figure 4:**
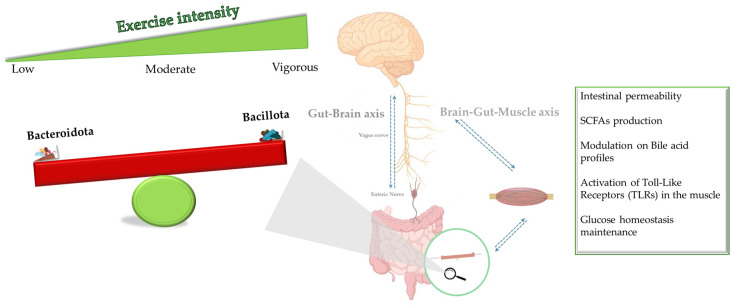
Variation in the Bacillota/ Bacteroidota ratio can occur based on physical intensity training and allow, in this way, our body to respond to intense exercise with several physiological and metabolic mechanisms.

Here, a meta-analysis was conducted using ten studies as the focus, based on the findings from the narrative reviews that were already available or from other systematic review reviews. As previously reported in the literature, increasing exercise training was observed to affect the composition of the gut microbiota, particularly decreasing Bacteroidota (formerly Bacteroidetes), and increasing Bacillota (formerly Firmicutes), indicating a shift in the B/B ratio [62,63,64]. The B/B ratio tended to enhance in subjects with high-intensity levels (vigorous, 60–84% VO_2_R), as suggested in previous works [62,63,64]. The Bacillota/Bacteroidota (B/B) ratio, which is often used to describe gut health, is one of the indicators considered in this study. In fact, in some studies, results have described the B/B ratio and shown that it undergoes variations in athletes versus sedentary individuals, proposing it as a possible biomarker of alteration of the health status of the gut [22]. According to the previous study, it was illustrated that the B/B ratio tends to decrease in subjects with sedentary or low physical activity [5]. Bacteroidota significantly decreased in response to the intensity of exercise, according to our findings. This change implies that this phylum, which is an essential component of the gut microbiota, may decline because of exercise therapies. It is well known that bacteria play a role in the complicated digestion of carbohydrates and the consumption of energy. The decline that is seen following exercise initiates the question of what influences PA specifically on this group and its implied metabolic counter; hence, diet modification is necessary. Contrary to expectations, Bacillota, a prominent phylum of gut microbiota, had a noteworthy rise following physical activity. This rise implies that exercise therapies may have a positive effect on Bacillota in the gastrointestinal tract. The gut metabolic processes may be linked to an increase in the number of bacteria that play a role in energy birth and storage. The ability to engage in endurance exercise indirectly strengthens the intestinal barrier. The gut barrier is indirectly strengthened by endurance exercise capacity. *Lactobacillus* (Bacillota) and *Bifidobacterium* have been demonstrated repeatedly to improve intestinal barrier integrity through a variety of methods, including greater capacity for butyrate synthesis. As a result, there is less migration of endotoxins and germs from the intestinal lumen into the systemic circulation, which lowers inflammation and strengthens immunity. The release of pro-inflammatory mediators, including lipopolysaccharide (LPS) and tumor necrosis factor-alpha (TNF-α), has demonstrated that healthy gut microbiota release a small quantity of LPS into the bloodstream, which is vital for the maintenance and development of the host immune system, and different pathophysiological reactions within various organs, including adipose tissue, the liver, and the endothelium [65,66]. Furthermore, the anti-inflammatory effects, which are typically accompanied by the release of cytokines and peptides, also exert an influence on skeletal muscles [67]. Thus, it is plausible that the increase of *Lactobacillus* enhances the integrity of the intestinal barrier and improves aspects of host health that have been associated indirectly with increasing the capacity for endurance exercise. The significant rise that is seen in response to exercise emphasizes the beneficial role that PA plays in regulating this phylum, possibly promoting greater overall health and metabolic balance. Furthermore, the Bacillota phylum encompasses genera that are capable of producing short-chain fatty acids (SCFAs) [67,68]. Short-chain fatty acids (SCFAs) are widely recognized for their involvement in crucial processes such as maintaining colonocyte integrity, enhancing barrier function, and increasing mucin expression through the regulation of specific gene expression [68,69]. Moreover, the scientific evidence supports the notion of an inverse relationship between the type and intensity of physical activity and the quantity of fecal bile acids that influence the increase in the Bacillota (mainly *Clostridia* spp.) [70]. Collectively, these observations reinforce the concept that gut bacteria play an active role in metabolic homeostasis (Figure 4) [11,71].

This systematic review and meta-analysis have certain limitations. First off, the sociodemographic traits and dietary practices of the participants in the selected studies varied significantly, which restricts their comparability and may have an impact on the consistency of the findings. In addition, the studies analyzed varied in duration and frequency, which limited their comparability and the characterization of the effects of time and exercise volume. There were variations in the study’s quality as well, but the main problem with the quality and the robustness of the conclusions was the absence of controls over confounding variables. However, this systematic review and meta-analysis offers new perspectives for future research into the changes in bacterial composition and is a first step in meticulously characterizing the different forms of biodiversity observed in athletes’ gut microbiota.

## 5. Conclusions

The available data indicate that the microbiota can be affected by the level of physical intensity, with implications for bacterial abundance and diversity indices. These findings have implications for the physiological and metabolic system. Our meta-analysis indicates that the Bacillota/Bacteroidota ratio tends to increase in relation to levels of physical activity intensity. This suggests that Bacillota may play a specific role in adaptation to higher levels of physical activity. In future, specific study of the role of individual species could lead to intervention strategies aimed at stimulating beneficial pathways for athletes. It is therefore imperative to increase our knowledge of the interaction between the gut microbiota and physical activity.

## Figures and Tables

**Table 1:** Characteristics of original studies and main results related to physical activity and microbiota (in animals). ND = Not described.

Author, Year, Country, Ref	Study Design	Subjects (Animals)	Sample Size	Age (Mean Value ± SD, Range/Age Group %)	Gender	Type of Sample	Type of Exercise	Duration, Frequency, Training Volume	Outcome	Quality Assessment (NOS Scale)
Pekkala, 2017, Finland [42]	Cohort study	Mice	20	7 weeks	Male and female	Fecal	Endurance exercise treadmill running	ND	Intrinsic aerobic capacity governs the microbiome, which may influence body weight, metabolism, and gene expression	8 (Newcastle–Ottawa Quality Assessment Scale—Cohort Studies)
Yang, 2021, China [44]	Cohort study	Mice	30	5 weeks old	Male	Fecal	Moderate intensity treadmill exercise	3 days a week, 10–20 min a day, slope 0, speed 10–13 mile per minute	Moderate-intensity treadmill exercise significantly increased the exercise capacity of mice and alters the core bacteria and bacterial metabolic activities	6 (Newcastle–Ottawa Quality Assessment Scale—Cohort Studies)
Mach, 2021, France [45]	Cohort study	Horse	20	10 ± 1.69 years	Male and female	Fecal, Blood	Endurance exercises	120 and 160 km race	Targeting the gut-mitochondria axis, therefore, appears to be a possible strategy to enhance athletic performance	6 (Newcastle–Ottawa Quality Assessment Scale—Cohort Studies)
Tung, 2019, Taiwan [46]	Cohort study	Mice	100	7 weeks old	Male	Fecal	Exhaustive swimming	Exhaustive swimming test, with 5% body weight (BW) loading on the tail	The mice with various intrinsic exercise capacities have different gut microbiome as well as transcriptome and proteome of soleus muscle by applying multi-omics approaches. The main bacteria and controlling factors, including miRNA and functional proteins, may be too much correlated with the adoption of physiological functions and exercise capacity	6 (Newcastle–Ottawa Quality Assessment Scale) Cohort Studies

**Table 2:** Characteristics of original studies and main results related to physical activity and microbiota (in humans).

Author, Year, Country	Study Design	Sample Size	Age (Mean Value ± SD, Range/Age Group %)	Gender	Type of Exercise	Duration, Frequency, Training Volume	Outcome	Quality Assessment
Petersen, 2017, USA [43]	Cross-sectional study	33	19–49	Male and female	Cycling	All participants spent a minimum of 6 h exercising per week	No significant correlations were found between taxonomic cluster and being either a professional or amateur level cyclist, high abundance of the genus *Prevotella* (≥2.5%) was substantially correlated with time noted for exercising during an average week	5 (Newcastle–Ottawa Quality Assessment Scale) Adapted for cross-sectional studies
Karl, 2017, USA [47]	RCT	73	>18	Male and female	Fifty-one kilometer of cross-country ski-march	4 days artic military training (51-km cross-country ski-march, during which volunteers skied in the 50:10-min work-to-rest ratios also carrying a ~45-kg pack)	Physiological stress is associated with the intestinal permeability, changes in gut microbiota by modifying diet and stress level can change intestinal permeability	3 (Cochrane risk-of-bias tool for randomized trials)
Yang, 2017, China [48]	Cross-sectional study	71	19–49	Female	ND	2-min warm-up at 50 W to access VO_2_ max	Cardiorespiratory fitness is associated with gut microbiota composition, independent of age and carbohydrate or fat intake	6 (Newcastle–Ottawa Quality Assessment Scale) adapted for cross-sectional studies
Tabone,2021, France [49]	Cohort study	40	18–50	Male	Cross-country running	10-min warm-up of continuous running on a treadmill at 60% of their maximum heart rate. After the warm-up, they ran with a slope of 1% at a speed of 10 km/h, with increase of 0.3 km per hour every 30 s until volitional exhaustion	The performance of a single exercise bout in cross-country non-professional athletes produces significant changes in the microbiota, in serum and fecal metabolome, which may have health implications	8 (Newcastle–Ottawa Quality Assessment Scale—Cohort Studies)
Oliveira, 2021, Portugal [50]	Cohort study	17	18–25	Female	Football	Perceived exertion method	The physical and physiological requirements of training and matches of an official international tournament did not vary the gut microbiota balance of elite female football players	6 (Newcastle–Ottawa Quality Assessment Scale—Cohort Studies)
Craven, 2021, Australia [51]	Cohort study	14	18–25	Male and female	Middle distance running	Three weeks of the normal training; 3 weeks of high-volume training (10, 20 and 30% increment in training volume during every successive week from NormTr); one-week taper (TaperTr; 55% exponential decrease in training volume from third HVolTr week)	The alpha-diversity and global formation of the gut microbiome were unaffected by differences in training volume. However, an increment in training volume led to various changes at the lower taxonomy levels that did not come back to pre-HVolTr levels following the taper period	8 (Newcastle–Ottawa Quality Assessment Scale—Cohort Studies)
Moitinho-Silva, 2021, Germany [52]	RCT	42	20–45	Male and female	Strength exercise, endurance exercise	Control group (general physical activity), Endurance group (at least 30 min of running three times per week), and Strength group (three days per week of whole-body hypertrophy strength training in the gym). The participants warmed up for five minutes on the treadmill, ergometer, or rowing machine before beginning their 30-min training session. One session included six distinct exercises, two for each leg, chest, and back, to develop the big and main muscle groups. The participants did one warm-up set (which was supposed to be 50% of the load set weight) and one load set for each activity. The weight for the load set was chosen so that eight repetitions were possible. If more than eight repetitions are doable on two consecutive training days.	Various types of exercise have different but balanced effects on the overall physiology of humans and very versatile microbial changes in the gut	4 (Cochrane risk-of-bias tool for randomized trials)
Han, 2020, China [53]	Cohort study	19	12–26	Female	Rowing	NA	Gut microbial communities depend upon the type of exercise associated with dietary factors and physical characteristics	7 (Newcastle–Ottawa Quality Assessment Scale—Cohort Studies)
Liang, 2019, China [54]	Cross-sectional study	31	20–24	Male and female	Professional martial arts	Higher-level group: exercise load (hours/week): 29.25 ± 9.48; lower-level group: 16.63 ± 6.82	The higher-level athletes had increased diversity and higher metabolic proportions of the gut microbiome for it may positively impact athletic performance	7 (Newcastle–Ottawa Quality Assessment Scale) adapted for cross-sectional studies
Cronin, 2018, Ireland [55]	RCT	90	18–40	Male and female	Aerobic and resistance exercise training	Eight-week combined aerobic and resistance exercise training program	The improved body composition with exercise is not depending on basic changes in the diversity of microbial populations in the gut. The various microbial characteristics already observed in long-term habitual athletes may be a late response to exercise and fitness enhancement	5 (Cochrane risk-of-bias tool for randomized trials)
Kulecka, 2020, Poland [56]	Cross-sectional study	71	14–72	Male and female	Marathon running; cross-country skiing	Highest level of training endurance sports athletes trained on average 1.58 ± 0.58 times per day, taking 7.25 ± 2.17 training units in one week lasting 2.79 ± 1.74 h per day on an average	Excessive training is involved with the changes in composition and elevation of increased bacterial diversity	6 (Newcastle–Ottawa Quality Assessment Scale) adapted for cross-sectional studies
Rettedal,2020, New Zealand [57]	Cross-sectional study	29	20–45	Male	HIIT training	Three weeks of high-intensity training (8–12 × 60 s cycle ergometer bouts at VO_2_ maximum power output interspersed by 75 s rest, three times per week)	The gut microbiome’s overall composition is not significantly altered by short-term HIIT, however some microbiome genera are linked to insulin sensitivity measures, and HIIT enhanced these markers in overweight subjects	5 (Newcastle–Ottawa Quality Assessment Scale) adapted for cross-sectional studies
Estaki, 2016, Canada [58]	Cross-sectional study	39	18–35	Male and female	Not specific	NA	Cardiorespiratory fitness is correlated with increased microbial diversity in healthy humans and that the associated changes are anchored around a set of functional cores rather than specific taxa	6 (Newcastle–Ottawa Quality Assessment Scale) adapted for cross-sectional studies
Jang, 2019, Korea [59]	cross-sectional study	45	25 ± 3 (bodybuilders); 20 ± 1 (distance runners) Healthy men 26 (±2) years	Male	Bodybuilding; distance running	NA	Athlete type was significantly associated with the relative abundance of gut microbiota at the genus and species level: *Faecalibacterium*, *Sutterella*, *Clostridium*, *Haemophilus*, and *Eisenbergiella* were increased (*p* < 0.05) in bodybuilders, while *Bifidobacterium* and *Parasutterella* were decreased (*p* < 0.05). At the species level, intestinal beneficial bacteria widely used as probiotics (*Bifidobacterium adolescentis* group, *Bifidobacterium longum* group, *Lactobacillus sakei* group) and those were generating the short-chain fatty acids (*Blautia wexlerae*, *Eubacterium hallii*) were decreased in bodybuilders and the increased in controls	6 (Newcastle–Ottawa Quality Assessment Scale) adapted for cross-sectional studies
Barton, 2021, Ireland [60]	Cohort study	2	31.5	Male	Marathon and triathlon	Regular aerobic exercise complimented with twice weekly resistance training	Sustained fitness improvements support alterations to gut microbiota and physiologically relevant metabolites	6 (Newcastle–Ottawa Quality Assessment Scale—Cohort Studies)

**Table 3:** Characteristics of samples and specificities of included studies in meta-analysis.

Author, Country, Year [Ref]	Biodiversity Indicators, Alpha Diversity	Biodiversity Indicators, Beta Diversity	B/B Ratio	Bacillota	Bacteroidota	Diversity	Comparison between Groups	Bacillota/Bacteroidota	Type of Exercise and Control	Classification of Exercise and Sport Intensity Based VO_2_ Max [38] and Details		Classification of Exercise and Sport Intensity Based METs Values [39] and Details	
								%		CON	PA	CON	PA
Craven, 2021, Australia [51]	↔Alpha-diversity (Shannon index, Chao1)	Microbial communities are similar	(A) CON = 1.3; PA = 0.7; (B) CON = 1.2; PA = 0.8	High-volume training: ↓ *Lachnoclostridium* (*p* = 0.02), *S. parasagunis* (*p* = 0.02) ↑ *R. callidus* (*p* = 0.03) *Lachnoclostridium* (bacillota) 21% *H. parainfluenzae* (pseudomonadota) 0.5% *S. parasanguinis* (bacillota) 3% *R. callidus* (bacillota) 15%	NA	Only three types of Bacillota identified including *Lachnoclostridium*, *S. parasanguinis* and *R. callidus*. No bacteroidota present. Overall in Bacillota only *R.callidus* percentage increased after high volume and taper training as compared to normal training while *Lachnoclostridium* and *S. parasanguinis* percentage decreased in high volume and taper training	Faecal samples were obtained before and instantly after each training (three weeks of the normal training, three weeks of the high-volume training and a one-week taper training)	*Pasteurellaceae* (pseudomonadota) 0.5% *Haemophilus* (pseudomonadota) 0.5% *Lachnoclostridium* (bacillota) 21% *H. parainfluenzae* (pseudomonadota) 0.5% *S. parasanguinis* (bacillota) 3% *R. callidus* (bacillota) 15%	Middle distance running CON = Normal training; PA = Higher level training	(A) Vigorous (60–84% VO_2_R) (B) Vigorous (60–84% VO_2_R) + Normal Training volume	(A) Vigorous (60–84% VO_2_R) (B) Vigorous (60–84% VO_2_R) + Training volume increasing during the activity	(A) Vigorous > 6 METs; (B) Vigorous > 6 METs	(A) Vigorous > 6 METs; (B) Vigorous > 6 METs
Cronin, 2018, Ireland [55]	α-diversity Shannon index changes were non-significant (no α-diversity value provided)	Microbial communities are statistically different. Bray-Curtis’s dissimilarity β-diversity changes were non-significant (no β-diversity value provided)	CON = 0.02; PA = 0.0015	*Borrelia hermsii* (Spirochaetota) 2.3% *Mycoplasma pneumoniae* (Tenericutes) 6.6%; *Streptococcus thermophilus* 0.002%	NA	In training group the Bacillota are *Streptococcus thermophillus* and *Lactobacillus lactis*. and in one training group Bacillota are only *Streptococcus thermophillus*	Fecal samples collected before and after 8 weeks intervention (exercise only group, exercise and protein supplement group and control group)	Only exercise group: *Borrelia hermsii* (Spirochaetota) 2.3%; *Mycoplasma pneumoniae* (Tenericutes) 6.6%; only protein group: *Streptococcus thermophilus* 0.002%	Aerobic and resistance exercise training CON = Sedentary; PA = exercise	Low (20–39% VO_2_R)	High (60–84% VO_2_R)	Vigorous > 6 METs (METs consumption related to daily life)	Vigorous > 6 METs
Estaki, 2016, Canada [58]	↑ * Shannon, Chao1, Simpson ↑ Alpha diversity (Chao, Shannon, Simpson)	Microbial communities are statistically different. Beta diversity (Bray–Curtis)	CON = 1.4; PA = 0.53	↑ * *Lachnospiraceae* ↑ * *Christensenellaceae*, ↑ * *Ruminococcaceae* ↑ * *Clostridiales*.(*p* = 0.026), *Roseburia*, *Lachnospiraceae*, *Erysipelotrichaceae*	NA		Fecal samples were collected in healthy adults before and after one week of VO_2_ testing	*Erysipelotrichaceae*, *Coprococcus*, *Roseburia*, *Adlercreutzia*, and unknown members of *Clostridiales*, *Lachnospiraceae*, and *Erysipelotrichaceae*.	CON = lower level exercise; PA = higher level exercise	Low (20–39% VO_2_R)	High (60–84% VO_2_R)	Vigorous > 6 METs (METs consumption related to daily life)	Vigorous > 6 METs
Han, 2020, China [53]	↑ * Shannon, Simpson ↑ Alpha diversity (Shannon, Simpson)	Microbial communities are statistically different. Beta diversity (Jac card, Unifrac), ↑ B/B	CON = 2.31; PA = 3.89	↑ * *Ruminococcaceae*, *Clostridiales*, *Faecalibacterium* and *Lachnospiraceae*.	↑ *	Bacillota included are *Ruminococcaceae*, *Clostridiales*, *Faecalibacterium* and *Lachnospiraceae*.	Fecal samples were collected from adult elite athlete’s (AE), youth elite athlete’s (YE), and youth non-elite athlete’s (YN) during two months period	The average relative abundances of Bacillota and *Proteobacteria* of AE (76.27% and 8.73%) and YE (64.7% and 10.69%) were higher than those of YN (58.12% and 8.01%), the moderate relative richness of *Bacteroidota* in YN (26.19%) was significantly increased than that of AE (11.41%) and YE (16.63%). *Ruminococcaceae_unclassified*, *Clostridiales_unclassified*, *Faecalibacterium* and *Lachnospiraceae_unclassified* were highest in the AE cohort, and *Bacteroides* and *Prevotella* were prominent in the YE cohort and YN cohort	Rowing CON = non elite athletes; PA = elite athletes	Moderate (40–59% VO_2_R)	Vigorous (60–84% VO_2_R)	Vigorous > 6 METs	Vigorous > 6 METs
Jang, 2019, Korea [59]	↓ * Shannon, OTUs, Chao1 Diversity between groups: → Alpha diversity (Chao1) ≠ Beta diversity (PC) Alpha and beta diversity remains same in both groups	Microbial communities are statistically different	(A) CON = 4.79; PA = 15.69; (B) CON = 4.79; PA = 3.96	*Faecalibacterium*, *Sutterella*, *Clostridium*, *Haemophilus*, and *Eisenbergiella* were increased in (*p* < 0.05) in bodybuilders, while *Bifidobacterium* and *Parasutterella* were the decreased (*p* < 0.05)	NA	The Bacillota compromised of *Blautia wexlerae*, *callidus*, *Faecalibacterium_uc Faecalibacterium prausnitzii*, *Clostridium innocuum Eubacterium hallii*, *Ruminococcus*, *Weissella confusa*, *Lactobacillus sakei*. The Bacteroidota compromised of *Bacteroides stercoris* and *Bacteroides caccae*	Fecal samples were obtained from sedentary men as control and from bodybuilders and distance runners	*Faecalibacterium*, *Sutterella*, *Clostridium*, *Haemophilus*, and *Eisenbergiella* were the highest (*p* < 0.05) in bodybuilders, while *Bifidobacterium* and *Parasutterella* were the lowest (*p* < 0.05). At the species level, intestinal beneficial bacteria widely used as probiotics (*Bifidobacterium adolescentis* group, *Bifidobacterium longum group*, *Lactobacillus sakei* group) and those producing short-chain fatty acids (*Blautia wexlerae*, *Eubacterium hallii*) were the lowest in bodybuilders and the highest in controls	Body building; distance running (A) CON = not exercise; PA = bodybuilding; (B) CON = not exercise; PA = distance running	(A) Low (20–39% VO_2_R) (B) Vigorous (60–84% VO_2_R)	(A) Vigorous (60–84% VO_2_R) (B) Vigorous (60–84% VO_2_R)	(A) light < 3 METs (B) Vigorous > 6 METs	(A) Vigorous > 6 METs (B) Vigorous > 6 METs
Karl, 2017, USA [47]	↑ * Shannon, Chao1 ↑ Alpha diversity (Shannon) → Alpha-diversity (Chao1, OTU) ↑ B/B ratio	Bray-Curtis distances	CON = 1.28; PA = 1.86	*Pepto-streptococcus*, *Christensenella*, *Faecalibacterium*, *Staphylococcus*, unassigned taxa within the *Mogiobacteriaceae*, *Christensenellaceae*, *and Planococcaceae families*. *Verrucomicrobia* *, *TM7* *, *Tenericutes* *, *Spirochaetes* *, *Proteobacteria*, *Lentisphaerae* *, *Fusobacteria* *, *Bacillota* *, *Euryarchaeota* *, *Cyanobacteria*, *Bacteroidota* *, *Actinobacteria*	↓ *	↑ *	Fecal samples were collected from subjects the control group, the protein supplemented group and carbohydrate supplemented group two days before and one day after the stress exercises	*Pepto-streptococcus*, *Christensenella*, *Faecalibacterium*, *Staphylococcus*, unassigned taxa within the *Mogiobacteriaceae*, *Christensenellaceae*, and *Planococcaceae* families. *Verrucomicrobia* *, *TM7* *, *Tenericutes* *, *Spirochaetes* *, *Proteobacteria*, *Lentisphaerae* *, *Fusobacteria* *, *Bacillota* *, *Euryarchaeota* *, *Cyanobacteria*, *Bacteroidota* *, *Actinobacteria*	Fifty-one kilometer of cross-country ski-march CON = pre-training; PA = post-training	Vigorous (60–84% VO_2_R) [61]	Vigorous (60–84% VO_2_R) [61]	Vigorous > 6 METs	Vigorous > 6 METs
Kulecka, 2020, Poland [56]	Chao1, Simpson (*p* = 0.025 & *p* = 0.00059) ↑ Alpha diversity (Shannon, Simpson, Chao1), ↑ B/B ratio	Microbial communities are statistically similar	CON = 1.83; PA = 10.04	*Bacteroides*, *Prevotella*, *Alistipes*, *Sutterella* and *Subdoligranulum*, *Ruminococcaceae* family and *Barnesiella* genus, *Bacillota; Clostridia*; Clostridiales; *Lachnospiraceae; Lachnoclostridium; Proteobacteria; Gammaproteobacteria; Pasteurellales; Pasteurellaceae; Haemophilus; Bacteroidia; Bacteroidales; Prevotellaceae; Prevotella_9*	*Bacteroidota* ↑ * *Prevotella*		Stools samples were obtained from the 14 marathon runners, 11 cross country skiers and the 46 inactive healthy controls	*Bacteroides*, *Prevotella*, *Alistipes*, *Sutterella* and *Subdoligranulum*, *Ruminococcaceae* family and *Barnesiella* genus, *Bacillota*; *Clostridia*; *Clostridiales; Lachnospiraceae; Lachnoclostridium; Proteobacteria; Gammaproteobacteria; Pasteurellales; Pasteurellaceae; Haemophilus; Bacteroidia; Bacteroidales; Prevotellaceae; Prevotella_9*	Marathon running; cross-country skiing CON = lower level exercise; PA = higher level exercise	Low (20–39% VO_2_R)	Vigorous (60–84% VO_2_R)	(A) light < 3 METs	Vigorous > 6 METs
Liang, 2019, China [54]	↑ * Shannon (*p* = 0.019), Simpson(*p* = 0.001)	Microbial communities are statistically different	CON = 1.9; PA = 0.568	↑ * *Parabacteroides* (*p* < 0.001) *Oscillibacter* (*p* = 0.026 *Bilophila* (*p* = 0.036) *Megasphaera* (*p* = 0.04) *Phascolarctobacterium* (*p* = 0.028)	↑ * Megasphaera (*p* = 0.04)	*Veillonellaceae*, *Phascolarctobacterium*, *Oscillibacter* and *Megasphaera.* The *Bacteroidota* are *Porphyromonadaceae* and *Parabacteroides*	Fecal samples were collected from 12 large scale and 16 small scale athletes	Higher level group: *Porphyromonadaceae* 4.4%; *Veillonellaceae* 0.9%; *Parabacteroides* 2.3%; *Phascolarctobacterium* 2.2%; *Oscillibacter* 0.7%; *Megasphaera* 0.008%; *Bilophila* 0.3%; Lower-level group: *Porphyromonadaceae* 2%; *Veillonellaceae* 4.5%; *Parabacteroides* 0.8%; *Phascolarctobacterium* 0.5%; *Oscillibacter* 0.3%; *Megasphaera* 0.069%; *Bilophila* 0.075%	Professional martial arts CON = Lower level group; PA = Higher level group	Moderate (40–59% VO_2_R) + Lower-level of Exercise load (hours/week)	Moderate (40–59% VO_2_R) + Higher level of Exercise load (hours/week)	Vigorous > 6 METs	Vigorous > 6 METs
Oliveira, 2021, Portugal [50]	↑ * Shannon, Chao1, Simpson (*p* = 0.013)	Microbial communities are statistically different	CON = 1.78; PA = 1.677	↑ * *Collinsella aerofaciens* ↑ * *Faecalibacterium prausnitzii*	↑ * *Prevotella* *Copri* (31%)	*Bacillota* included are *Fecalibacterium* and *Bacteroidota* included is *Prevotella*	Fecal samples were collected from subjects on 2nd, 3rd, 9th and 10th day of matches	At the beginning, 50% of bacteria were Bacillota (Frimicutes) followed by Bacteroidota (28%) and Actinobacteria (19%). Bacillota (Frimicutes) (52%) was the most prevalent type overall at the conclusion of the event followed by Bacteroidota (31%) and Actinobacteria (14%). At baseline, Faecalibacterium (29%) was the most prevalent bacterial genus followed by Collinsella (16%) and Prevotella (13%). At the conclusion of the competition Faecalibacterium was the most prevalent overall (29%) followed by Prevotella (17%) and Collinsella (12%). Faecalibacterium prausnitzii was the most prevalent by the end of the competition (30%) followed by Collinsella aerofaciens (13%) and Prevotella copri (12%)	Football CON = Baseline; PA = End of study	Moderate (40–59% VO_2_R)	Vigorous (60–84% VO_2_R)	Vigorous > 6 METs	Vigorous > 6 METs
Petersen, 2017, USA [43]	↑ * Shannon (*p* = 0.0004), OTUs	Microbial communities are statistically different	CON = 2.05; PA = 2.33	NA	↑ * *Prevotella*		Fecal samples were obtained from 33 cyclists with extensive medical issues and no antibiotic use within the last year	NA	Cycling PA = professional level (the highest level) and CON = amateur level	Vigorous (60–84% VO_2_R) + lower level of Exercise load (h/week)	Vigorous (60–84% VO_2_R) + higher level of Exercise load (h/week)	Vigorous > 6 METs	Vigorous > 6 METs

↑ = increase,↑* = statistically significant increase, ↓ = decrease, ↓* = statistically significant decrease, ↔ = no differences between groups, CON = control, PA = physical exercice, NA = not available.

## Supplementary Materials

The following supporting information can be downloaded at: https://www.mdpi.com/article/10.3390/sports12080221/s1, Table S1. PRISMA Checklist. Figure S1. The funnel plot shows the distribution of studies for publication bias.
