# Supplementary figures and images for: The Role of Gut Microbiota in Different Types of Physical Activity and Their Intensity: Systematic Review and Meta-Analysis
Source: Sports (Basel). 2024 Aug 14;12(8):221. doi: 10.3390/sports12080221 (PMC11360093; doi:10.3390/sports12080221)

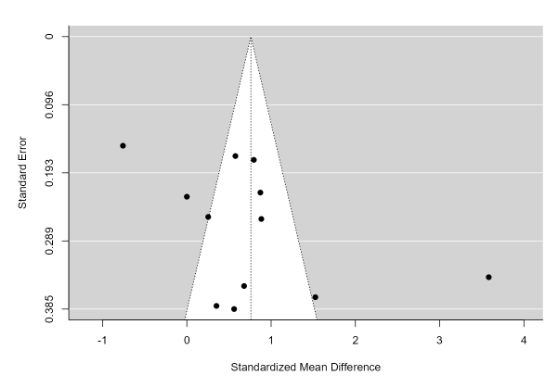

Supplement: Supplementary file 1 [file sports-12-00221-s001.zip › Figure S1.png]
